# Hormone Involvement in Tissue Development, Physiology and Oncogenesis: A Preface to the Special Issue

**DOI:** 10.3390/cancers12092642

**Published:** 2020-09-16

**Authors:** Claudio Luparello

**Affiliations:** Department of Biological, Chemical and Pharmaceutical Sciences and Technologies, University of Palermo, Viale delle Scienze, Edificio 16, 90128 Palermo, Italy; claudio.luparello@unipa.it; Tel.: +39-091-238-97405

Hormones, i.e., the products of specialized endocrine cells which spread throughout the body via the bloodstream, control the normal development and growth of organisms at the embryo-fetal stage and, in adult life, regulate, integrate, and coordinate a range of different physiological processes which concern virtually all body tissues. They exert their biological effects by interacting with either surface or intracellular receptors, thereby activating signalization pathways [1]. For example, steroid hormones, such as those released by the adrenal glands, testes and ovaries, once freely crossed through the plasmalemma, bind to receptors that act as ligand-dependent transcriptional regulators and influence the expression of a plethora of target genes responsible for diversified biological responses, including sexual differentiation, osmoregulation, metabolism and developmental roles in various fetal systems among others [2,3]. On the other hand, lipid-insoluble hormones, such as those released by hypophysis, parathyroids and the endocrine pancreas, follow different transduction pathways. One possibility is that they can recognize membrane receptors in the target cytotypes and stimulate the activation of effector systems, i.e., diversified and specific signalizations, mediated by intracellular second messengers which amplify the stimulus into a cascade of biochemical events, leading to final cell responses. Alternatively, these hormones can bind to protein kinase receptors which, once switched on, initiate, by themselves, the phosphorylation process of a variety of intracellular targets, ultimately modifying a cell’s physiological state [4,5]. Given that single hormones can utilize more than one system and elicit multiple responses from one cell, and that different cytotypes respond in a variety of manners to the same hormone, while also considering the occurrence of co-stimulations and the co-participation of other growth factors/cytokines and microenvironmental components, an enormous and ever-growing amount of in vitro and in vivo data have been published which unveil selected aspects of this complicated scenario that still has plenty of unexplored points.

The same complexity applies for the involvement of hormones in cancer development. It is well-known that excessive or impaired hormonal activities have been proven to increase the risk of oncogenesis in various tissues acting on both the initiation and the promotion stages of cancer etiology. Suffice it to say, just to cite two examples, that estrogen over-production may induce endometrial hyperplasia, which has a propensity to evolve towards endometrial cancer, or that *BRCA1* mutation in breast tissue is associated with deregulation in estrogen and progesterone receptor expression and functions, potentially leading to breast cancer development [6,7]. Moreover, single hormonal molecules can play different, sometimes even opposite, roles on paired normal and tumoral cytotypes (e.g., [8,9]). In addition, as a result of their de-differentiation, tumor cells themselves can ectopically release hormones and induce the occurrence of paraneoplastic syndromes which can determine the abnormal behavior of organ systems and lead to permanent damage [10]. 

In light of the massive scientific and research interest in a deeper knowledge of the biomolecular and physio-pathological features of hormone involvement in tissue functioning/derangement, for this Special Issue, researchers are invited to contribute original articles or reviews that report and discuss either their latest findings or literature data from a 360 degree perspective about the epi/genetic, biochemical, physiological and pharmacological effects of hormones on developing, developed and onco-transformed tissues, with a particular, although not exclusive, focus on the comparative analysis of paired tumoral and normal histotypes which might unveil novel metabolic reprogramming routes and potential prognostic biomarkers. 
